# Determining of Factors Influencing the Success and Failure of Hospital Information System and Their Evaluation Methods: A Systematic Review

**DOI:** 10.5812/ircmj.11716

**Published:** 2013-12-05

**Authors:** Farahnaz Sadoughi, Khalil Kimiafar, Maryam Ahmadi, Mohammad Taghi Shakeri

**Affiliations:** 1 Department of Health Information Management, School of Health Management and Information Sciences, Iran University of Medical Sciences, Tehran, IR Iran; 2 Department of Medical records and Health Information Technology, School of Paramedical Sciences, Mashhad University of Medical Sciences, Mashhad, Iran; 3 Department of Community Medicine, Faculty of Medicine, Mashhad University of Medical Sciences, Mashhad, IR Iran

**Keywords:** Hospital Information Systems, Health Information Systems, Review Literature

## Abstract

**Background:**

Nowadays, using new information technology (IT) has provided remarkable opportunities to decrease medical errors, support health care specialist, increase the efficiency and even the quality of patient’s care and safety.

**Objectives:**

The purpose of this study was the identification of Hospital Information System (HIS) success and failure factors and the evaluation methods of these factors. This research emphasizes the need to a comprehensive evaluation of HISs which considers a wide range of success and failure factors in these systems.

**Materials and Methods:**

We searched for relevant English language studies based on keywords in title and abstract, using PubMed, Ovid Medline (by applying MeSH terms), Scopus, ScienceDirect and Embase (earliest entry to march 17, 2012). Studies which considered success models and success or failure factors, or studied the evaluation models of HISs and the related ones were chosen. Since the studies used in this systematic review were heterogeneous, the combination of extracted data was carried out by using narrative synthesis method.

**Results:**

We found 16 articles which required detailed analysis. Finally, the suggested framework includes 12 main factors (functional, organizational, behavioral, cultural, management, technical, strategy, economy, education, legal, ethical and political factors), 67 sub factors, and 33 suggested methods for the evaluation of these sub factors.

**Conclusions:**

The results of the present research indicates that the emphasis of the HIS evaluation moves from technical subjects to human and organizational subjects, and from objective to subjective issues. Therefore, this issue entails more familiarity with more qualitative evaluation methods. In most of the reviewed studies, the main focus has been laid on the necessity of using multi-method approaches and combining methods to obtain more comprehensive and useful results.

## 1. Background

Nowadays, using new information technology (IT) has provided remarkable opportunities to decrease medical errors, support health care specialist, increase the efficiency and even the quality of patient’s care and safety (1, 2). On the other hand, there are numerous problems in the scope of IT-based systems in the field of health care; therefore, it causes a deep gap between the positive potential for IT to help health care organizations and their negative impacts. It means that a huge amount of money is invested in health information systems, but a significant portion of this money is wasted for inefficient systems or not implemented ones (3).

Evaluation means “the act of measuring or exploring properties of a health information system (in planning, development, implementation, or operation), the result of which informs a decision to be made concerning that system in a specific context” (4). An evaluation which is carried out based on suitable investment and approved techniques can cause the organization to have a forward movement (5, 6). Organizations require a comprehensive evaluation framework, which can help create and develop methods of information system evaluation (7); on the other hand, the identification of methods for information systems evaluation can be possible through the identifying the success and failure factors of these systems (8).

When we talk about success, we should identify what the criteria and parameters used for the evaluation of success are. Success is considered as a dynamic concept (9). Whether the system achieves its intended purpose from its establishment is what we mean by success; moreover, it should be carried out based on an anticipated time table and budget, while the project team and its users are satisfied with the results and this satisfaction should be constant (10). Since the information system is complicated and multidimensional, it may succeed or fail in different situations (11, 12). These days, due to increasing attention of different organizations to expenses related to the projects of information systems and gaining at least the minimum benefits from them, studying their success or failure has its special importance (13-15). There are various reports presented pertaining to the high rates of failure in IT projects in industrial sector and health care organizations especially in hospitals (3, 16-19). Kaplan in his study asserts that the rate of failure in implementation of IT in health care organizations of America is almost 50 percent (20).

Information systems are the combination of different elements, among which the measurement of some elements is easier than that of the others. The evaluation framework which is simply concentrated on elements whose measurement is easier cannot introduce a perfect framework in evaluation of information systems (21). Many case studies have been done, whose results are based on one or a few factors which affect the success of IT-based systems, so it mentions this important point that the findings of such literatures should be put together as a puzzle. In this case, a lot of benefits can be achieved from the significant studies done in this field by using different approaches (22). Just one comprehensive evaluation study can show whether a specific system can be successful in a special place or not. Moreover, each evaluation criterion must be measured through an appropriate method. A perfect evaluation should include all proper success factors (23, 24).

Previous researchers have discussed the challenges of the evaluation of health information systems and the problems resulting from lack of unique conceptual framework to guide evaluation researches (25, 26). The most significant challenge in evaluation of information systems used in health care is that information systems can influence the improvement of treatment and the patient’s health level. Due to this potential influence on the patient’s life, more accurate criteria should be applied for the evaluation of these systems (5, 11, 27). Of course, understanding the effects, results and prerequisites necessary for successful implementation of information technologies in health care require a multi-factor viewpoint (15).

Some success and failure factors have either less or more importance in different information systems (28). The main focus of our study is placed on the HIS, a computer system is designed to support the hospital needs for comprehensive information including patient’s information, clinical information and financial management (29, 30). The main purpose of this system is improvement the quality of care provided for patients (31, 32). Although the integrated HIS leads to more efficient use of sources, it has not resulted in effective service offering, improvement quality of care and increased productivity in many countries (11, 33). Unfortunately, the measurement methodologies of different effects of these systems have not been improved along with the development of these systems, while the future managers and users of HISs need accurate evaluation of these systems (11, 34).

## 2. Objectives

The purpose of this systematic review was the identification of the HIS success and failure factors and the evaluation methods of these factors. This study emphasizes the need to a comprehensive evaluation of HISs which considers a wide range of success and failure factors in these systems.

## 3. Materials and Methods

**Figure 1.**
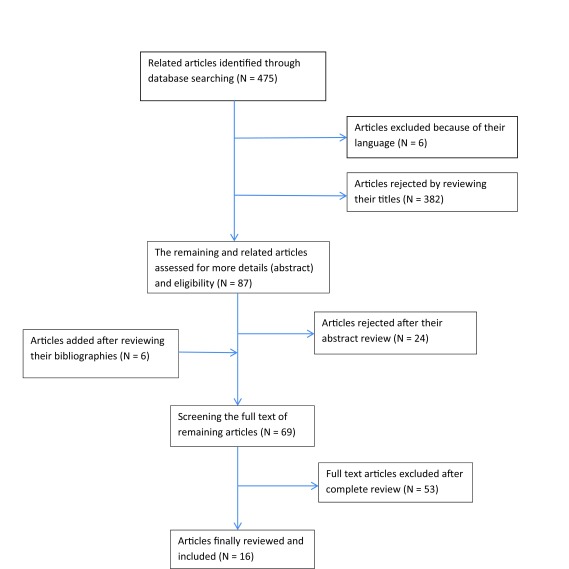
Flowchart of Search and Select the Included Articles in Systematic Review

We searched for relevant English language studies based on keywords in title and abstract, using PubMed, Ovid Medline (by applying MeSH terms), Scopus, ScienceDirect and Embase (earliest entry to March 17, 2012). In addition, we applied methods such as documentation review of relevant agencies (like Statistics and Information Technology Office in Ministry of Health and Medical Education) and databases that provide grey literature (like system for information on grey literature) for publication bias control. Also, searching was supplemented by scanning bibliographies from identified studies. The key journals have been manually searched to find references which may not be found in databases and the list of references. Table 1 represents the used search strategy and Figure 1 shows a flowchart representing the search and way of choosing studies. 

**Table 1.** Applied Search Strategy in Electronic Databases

Database	Search Strategy
**Ovid Medline**	1. exp Hospital Information Systems/
	2. exp Evaluation Studies as Topic/
	3. Evaluation Studies.pt.
	4. (fail or failure or success or succeed).ti,ab.
	5. or/2-3
	6. 1 and 4 and 5
	7. limit 6 to English language
**Pub Med**	
	1. "Hospital Information Systems"[Mesh]
	2. "Evaluation Studies as Topic"[Mesh] OR "Evaluation Studies" [Publication Type]
	3. fail[tiab] OR failure[tiab] OR success[tiab] OR succeed[tiab]
	4. English[la]
	5. and/1-4
**Embase**	
	1. exp Hospital Information Systems/
	2. exp evaluation/
	3. (fail or failure or success or succeed).ti, ab.
	4. and/1-3
**Scopus**	
	1. TITLE("hospital information system")
	2. TITLE-ABS-KEY(success OR succeed OR fail OR failure OR evaluation OR assessment OR evaluate OR assess)
	3. 1 and 2
	4. SUBJAREA(mult OR medi OR nurs OR vete OR dent OR heal)
	5. 3 and 4
**Science Direct**	
	1. TITLE-ABSTR-KEY("hospital information system")
	2. TITLE-ABSTR-KEY(success OR succeed OR fail OR failure OR evaluation OR assessment OR evaluate OR assess)
	3. 1 and 2

Two reviewers independently examined all titles and abstracts. Cohen’s Kappa coefficient was used to measure the inter-rater agreement on the inclusion or exclusion of the articles (k=0.77). The difference between the ideas of the two reviewers was settled by asking the idea of the third reviewer. To control assessment bias, reviewers were blinded about each other’s decisions and some information such as the names of journals and authors that could influence their decisions. Studies which considered models and success or failure factors, or studied the evaluation models of the HIS and the related ones were chosen. The studies examining the information systems in a specific area apart from the field of health care and in the field of public health as well as primary care in addition to non-English articles were excluded from the study.

Assessing the quality of qualitative research has attracted much debate and there is little consensus regarding whether quality can or should be assessed in relation to qualitative research (35-37). Since most articles which entered in this study were review articles and data analysis was done on the words and phrases in the articles using meta synthesis, to avoid losing valuable insights that existed in some studies that might be omitted in critical appraisal, we did not assess the quality of the selected studies.

Since the studies used in this systematic review were heterogeneous, the combination of extracted data was carried out by using narrative synthesis method. Finally, the common and varying aspects of the factors and their evaluation methods used in the selected studies were determined. Then these factors and methods were classified in different groups, and a framework was offered for the evaluation of success and failure factors in the HIS.

## 4. Results

Searching the online databases resulted in 475 articles. Initial screening of titles and abstracts rendered 63 articles eligible for further full-text review. Six additional articles were identified by reviewing the bibliographies, yielding a total of 69 articles. Based on reviewing the full-text of remaining articles, 53 articles were excluded since they did not correspond with the criteria and purpose of this study, and finally 16 articles remained which required detailed analysis. The summaries of findings pertaining to these articles are presented in Table 2. Most selected studies considered factors such as management (n = 11), behavioural (n = 15), organizational (n = 13) and economy (n = 11) as success factors (Table 3). Moreover, some evaluation methods such as interviews (n = 4), questionnaires (n = 5) and usability measurement methods (n = 7) were the most common methods in selected articles (Table 4). 

**Table 2.** The Summary of Findings Extracted From Selected Studies

Authors/ Year/ Reference No.	Study Methods	Success/ Failure Factors	Evaluation Methods/Approaches
**M.J. Van Der Meijden et al./ 2003 (23)**			
	Literature review	System Quality	
		Information quality	Questionnaires; Time studies; Work sampling
		User satisfaction	Open-end interviews; Questionnaires; Triangulation
		Usage	Work sampling; Time studies; Kept the log
		Individual and organizational impact	Chart review; Interviews; Work sampling; Questionnaires; Triangulation
**G. Pare et al./ 2008 (38)**	Literature review and a Delphi study	Risk dimensions and risk factors: Technological (introduction of a new technology; complex/unreliable technical infrastructure or network; complex software solution; complex/incompatible hardware; poor software performance); Human/user (unrealistic expectations; overall resistance to change; lack of cooperation/commitment from users; poor computer skills; prior negative experiences with Clinical Information System (CIS) projects); Usability (poor perceived system ease of use; poor perceived system usefulness; misalignment of system with local practices and processes); Project team (changes to membership on the project team; poor project leadership; lack of required knowledge or skills; lack of clear role definitions; negative attitude of project team members); Project (large and complex project; project ambiguity; changes to requirements; insufficient resources; lack of a project champion; lack of a formal project management methodology); organizational/ environmental (lack of commitment from upper management; organizational instability; lack of local personnel knowledgeable in IT; legal and ethical constraints; privacy and confidentiality issues); strategic/political (misalignment of actors’ and partners’ objectives and stakes; political games/conflicts; unreliable external partners)	Not mentioned
**P. Yu/ 2010 (39)**	Literature review and a case study	End user HIS perspectives	Cross sectional and longitudinal questionnaire
		Understanding how and why things have happened and would happen, and end users’ perceptions on what can be done better	Interview or focus group discussion
		Validate the changes in work practices associated with the introduction of the HIS	Work sampling with direct observational study
		Changes in quality of records associated with the introduction of the HIS	Auditing records that have been recorded both before and after the introduction of the HIS
**D. Zikos et al./ 2011 (40)**	Literature review and a case study	failure factors including: Underfunding; Inadequate use of standards; Lack of skilled IT experts; Insufficiently trained personnel and users’ reserve; Lack of a strategic plan; Lack of central planning; difficulties in the acceptance and incorporation of IT; Existing individual interests; Users reluctant to handle sensitive data; Difficulties in incorporating standards	Exploratory study; Feasibility study; Cost-productivity; Risk analysis
		Success factors including: Role of the hospital management (efficiently utilized skills of IS users; contributed to the IS planning; active participation); Education and training (training provided during the IS introduction; IT department providing active support); User support during the implementation (motivators were offered to the employees; external consultant (for IT department); management provided support to users); Contracted task agreement (contract-maintenance support provided; formal documentation system followed; criteria for system specs specified; revision-modification was agreed; schedule-deliverables agreed); Integrated complete information system; Technical specifications (maintenance -system scalability is ensured; specific use instructions are provided; data back-up mechanisms are supported; data security is adequate; IS is fast and flexible; The menus are localized); Standards and coding (HL7; DICOM; GMDN; ATC; ICD10); Evaluation of IS (patient satisfaction evaluation; employee satisfaction evaluation; measurement of cost-productivity)	
**MM Yusof et al./ 2008 (41)**	Literature review and a case study	Technology: System quality (data accuracy; data currency; database contents; ease of use; ease of learning; availability; usefulness of system features and functions; flexibility; reliability; technical support; security; efficiency; resource utilization; response time; turnaround time); Information quality (importance; relevance; usefulness; legibility; format; accuracy; conciseness; completeness; reliability; timeliness; data entry methods); Service quality (quick responsiveness; assurance; empathy; follow up service; technical support)	mix method (Quantitative and qualitative)
		Human:System use (amount/duration: (number of inquiries; amount of connect time; number of functions used; number of records accessed; frequency of access; frequency of report requests; number of reports generated); use by whom? (direct vs. chauffeured use); actual vs. reported use; nature of use (use for intended purpose; appropriate use; type of information used); purpose of use; level of use (general vs. specific); recurring use; report acceptance; percentage used; voluntaries of use; motivation to use; attitude; expectations/belief; knowledge/expertise; acceptance; resistance/reluctance; training) User satisfaction (satisfaction with specific functions; overall satisfaction; perceived usefulness; enjoyment; software satisfaction; decision making satisfaction)	
		Organization: Structure (type; size; culture; planning; strategy; management; clinical process; autonomy; communication; leadership; top management support; medical sponsorship; champion; mediator; teamwork); Environment (financing source; government; politics; localization; competition; inter-organizational relationship; population served; external communication); Net benefits (clinical practice (Job effects; task performance; productivity; work volume; morale); Efficiency; Effectiveness (goal achievement; service); Decision making quality (analysis; accuracy; time; confidence; participation); Error reduction; Communication; Clinical outcomes (patient care; morbidity; mortality); Cost)	
**V.P Aggelidis et al./ 2012 (42)**	Literature review and personal interviews with users	End user satisfaction; Information quality (accuracy; relevance; completeness; Currency; timeliness; format; security; documentation and reliability); Electronic Data Processing (EDP) staff and services (staff attitude; relationships; level of support; training; ease of access and communication); User knowledge or involvement (user training; user understanding and participation); System quality (speed; features; robustness and upgrade flexibility; user documentation); Interface quality (hardware devices; software and other telecommunications facilities); Service quality (the support provided by the information department; the support provided by the maintenance company)	Not mentioned
**L.A Hanmer/ 2007 (43)**	Literature review and a case study and interviews with local experts	Factors at hospital level (knowledge and understanding of Computerized Hospital information systems (CHIS); appropriateness of CHIS design; CHIS performance; availability of hospital resources for implementation; related training and ongoing support of the CHIS (‘hospital resources’)); Factors at provincial level (CHIS supplier knowledge and understanding of the environment; CHIS software fit with user requirements; organizational and contractual mechanisms such as customization and adaptation); Resource availability	Not mentioned
**L.W Peute et al./ 2010 (44)**	Longitudinal study with interviews, questionnaire, field and documentary historical methods	Success factors including: Political; strategy (transparency of vision; scope and objectives; top level commitment and higher level support); Managerial; economy; education (sufficient funds; internal communication and feedback; transparency staging of the implementation; flexible planning and strategy; requirements analysis; user needs analysis; multidisciplinary teamwork; end-user involvement; project evaluation; user-support during introduction; sufficient training before and during introduction) Technical; functional (workflow analysis; support clinical protocols; user centered design; usability; consistent; intuitive and user friendly interface; decision support; customization; flexibility; adaptability; system speed; available functionality; system maturity; system testing and evaluation; multi-dimensional integration) Cultural; behavioral; organizational (complexity of work practices; value to users; collaboration and trust; social relations; open attitude; culture of involved department; power; control and politics; organizational readiness; involvement of end-users; contacts); A feedback mechanism needs to be integrated into the software development cycle and implementation process; Reduce the complexity of the system implementation by dividing it up in separate issues; Project mechanisms to react on changes	Organizational readiness; Usability and ethnographic studies
**E-S Nahm et al./ 2007 (45)**	Literature review	User satisfaction	Questionnaire
		Clinical outcomes (patient clinical status; patient safety; length of stay; and mortality rates)	Randomized controlled trials (RCTs); Use proxy outcomes (decreased medication errors, improved adherence to practice guidelines, and improved quality of documentation)
		Financial impact	Return on Investment (ROI) analysis (benefit-to-cost ratio; Net Present Value (NPV); Break-even period or payback analysis)
		Outcomes of CIS implementation	Quantitative designs
			Experimental designs such as RCT
			Non-experimental designs
			Quasi-experimental designs (one group pre- and post-test studies, Time and motion studies)
			Descriptive design (Survey studies)
			Qualitative designs
			User testing (thinking-aloud method; observation; videotaping; and interviewing)
			Interviews (structured, semi-structured, or unstructured)
			Triangulation (use of multiple sources of data, observers, methods, or theories to draw conclusions)
**D.H Freed/ 2006 (46)**	Review article	Failure factors including: Not reengineering; No fun to use; Automation, not information; No structured systems development methodology; No user governance; Not user-friendly; Poor or no strategic alignment; No dedicated project resources; Questionable data integrity; Organizational and/or user instability	Not mentioned
**B. Kaplan/ 2009 (17)**	Literature review and workshop report	Success factors including: Technical issues related to functionality and interoperability; Social, cultural, and financial issues; Organizational, behavioral, and cognitive factors; Provide incentives; Remove disincentives; Identify and mitigate risk; Allow resources and time for training; Learning to input data; Learn from the past and from each other	Longitudinal and qualitative evaluation
		Failure factors including: Difficulties of communicating across different groups; The complexity of IT undertakings; The need to integrate all aspects of projects, work environments, and regulatory and policy requirements; The difficulty of getting all the parts and participants in harmony	
**B. Rahimi/ 2007 (15)**	Literature review	Changes in clinical practice behavior	Experimental designs
		Influences of the new systems on the organization and its personnel	Usability testing; Cognitive studies; Ethnography studies or socio-technical analysis
		Analyzing cost and benefits	Use subjective approaches combined with quantitative studies
		Research the systems’ outputs	Clinical trial or cohort study
		In general: have a multi-actor perspective in order to understand the effects, consequences and prerequisites that have to be achieved for the successful implementation; Organizational and economic factors; User and patient satisfaction	In general: The most common type of analytical approach used takes the form of a case study
**J. Brender et al./ 2006 (28)**	Analysis of the conference proceedings and a Delphi study	Success factors: Functional (careful preparation of the user requirements specification to appropriate and balanced take into account and express users requirements; needs as well as demands; alignment of the role and design of the IT-system; coping with the complexity; flexibility towards dynamic changes and changes in the organizational context; added functionality are provided by the IT-based system; enabling users to provide new or better services); Organizational (collaboration and cooperation; make implementation a transparent process within the organization; work from the workflow; high competences; support from higher level organizations); Behavioral (the users are key; the personal attitude; engagement and commitment; motivational activities); Cultural (understand medicine and healthcare in general as a separate culture; understand the local culture; preparedness and willingness towards cultural change); Political (high-level commitment; monitoring political implications; considering IT-systems a service rather than a product from a vendor; collaboration in providing new solutions; transparency); Management (management support; flexible planning; prospective and proactive control; consider IT implementation as a change process; coping with the impact of change; user involvement; strategy; communication; handling the diversity within stakeholder goals); Technical (standard based; data validity procedures are part of system qualities; use proven technology; usability; integrated functionality; communication standards; balance between flexibility and stability; evolution rather than revolution; flexibility and adaptability; enabling future functional and technical changes); Legal aspects (know what the legal constraints /opportunities are); Strategy (national; regional; organizational; accepted also at lower levels); Economy (there has to be a return of investment (whether material or immaterial); justification of increase of costs; sufficient funding); Education (sufficient training); User acceptance	Not mentioned
		Failure factors: Functional (the system does not meet expectations; limitations in the way the user can express his/herself; moving target); Organizational (not understanding the organizational context); Behavioral (overloading the user; underestimating user acceptance; resistance because of fear or loss of control of own job situation); Cultural (assuming that what works at one place also works somewhere else; users have too high expectations); Management (overambitious implementation plans; judgment based on wrong premises; improper tendering; business reorganization of the vendor); Technical (limitations in the way the user can express his/herself; the technology is so restricted that it impacts design and implementation choices; response rate and other performance measures; vendor did not support the functionality quoted; insufficient verification of conformity with requirements specification); Legal (low concern on regulations and standards; compliance with laws and existing ethical rules of conduct); Economy (lacking financial power of a vendor); Education (visible discrepancy between successive versions of the IT-based system)	
**VP Aggelidis/ 2008 (11)**	Literature review	The quality of information provided to users; The impact of IS on users’ thinking; decisions or actions; Organizational factors; Socio-technical factors	Technical verification and validations (during system development); Pilot and feasibility studies (after implementation); Monitoring studies (during routine use)
		Economic factors	Cost minimization analysis; Cost effectiveness analysis; Cost utility analysis; Cost benefit analysis
		System usage	Recording the length of user connection; The number of computer functions utilized; The number of client records processed; The number of tasks performed; The level of sophistication of usage; Questionnaire
		End-user satisfaction (EUS)	Not mentioned
**R. Heeks/ 2006 (3)**	Literature review and a case study	Fit between an organizational system, an information system, a management system, and its environment; Fit between technology and the task it is intended to support; Fit between IS and organizational strategy; Size of gap that exists between current realities and design conceptions of the HIS Professional, technical, economic and political factors; Designers and their cultural values, objectives; Assumptions about the users’ activities, skills, culture and objectives, and assumptions about the user organization’s structure, hardware and software infrastructure Information (data stores, data flows, etc.); Technology (both hardware and software); Processes (the activities of users and others); Objectives and values (the key dimension, through which factors such as culture and politics are manifest); Staffing and skills (both the quantitative and qualitative aspects of competencies); Management systems and structures, and other resources (particularly time and money) Archetypes of failure: Hard-soft gaps, Design-implementation context gaps, Public-private sector gaps, Country gaps	Design-reality gap model as a post hoc evaluation tool and as a pre hoc risk assessment and mitigation tool
**J. Brender et al./ 2004 (47)**	Workshop report	Not mentioned	Analysis of work procedure; Balanced scorecard; Delphi; Field study; Focus group interview; Heuristic evaluation; Interviews (non-standardized); Logical framework approach (LFA); Questionnaires (non-standardized); Risk assessment; Social network analysis; SWOT; Stakeholder analysis; Usability; Video recording; WHO: Framework for assessment of strategies; Clinical/Diagnostic performance; Cognitive assessment; Cognitive walkthrough; Technical verification; Functionality assessment; Randomized controlled studies; Effect assessment

**Table 3.** The HIS Success Factors and Their Frequency in Selected Studies

Success Factors	Sub Factors	Frequency (%)	Reference No.
**Functional**	Preparation of the user requirements	4 (25)	(38, 43, 44, 28)
	Alignment of the role and design of the HIS (Task-technology adaption)	4 (25)	(3, 28, 39, 41)
	Flexibility towards dynamic changes and changes in the organizational context	4 (25)	(28, 41, 43, 44)
	Added functionality are provided by the HIS, enabling users to provide new or better services	3 (18.75)	(28, 41, 44)
	Improve clinical performance and outcomes	4 (25)	(15, 41, 44, 45)
	In general	9 (56.25)	(3, 15, 17, 28, 38, 39, 41, 43, 44)
**Organizational**	Collaboration and cooperation	6 (37.5)	(17, 28, 38, 41, 42, 44)
	Participation in decision-making	3 (18.75)	(11, 41, 44)
	Work from the workflow	3 (18.75)	(28, 44, 46)
	Support from higher level organizations	3 (18.75)	(28, 41, 44)
	Make implementation a transparent process within the organization	2 (12.5)	(28, 44)
	Organizational stability	3 (18.75)	(38, 44, 46)
	Rate of hospital independence and authority	1 (6.25)	(41)
	Organizational capacity for changes	2 (12.5)	(39, 44)
	In general	13 (81.25)	(3, 11, 15, 17, 23, 28, 38, 39, 41-44, 46)
**Behavioral**	User involvement	4 (25)	(28, 42, 44, 46)
	User engagement and commitment	6 (37.5)	(28, 39-42, 46)
	Resistance to changes	3 (18.75)	(28, 38, 41)
	User knowledge and skills	6 (37.5)	(3, 38, 40-43)
	Stakeholder, user and patient satisfaction	15 (93.75)	(3, 11, 15, 17, 23, 28, 38-46)
	Motivational activities	5 (31.25)	(17, 29, 40, 41, 46)
	User acceptance (perceived system ease of use, perceived system usefulness)	7 (43.75)	(29, 38-43)
	In general	15 (93.75)	(3, 11, 15, 17, 23, 29, 38-46)
**Cultural**	Understand health care as a specific culture	1 (6.25)	(29)
	Understand the local culture (such as attention to cultural differences between public and private hospitals as well as developing and developed countries)	3 (18.75)	(3, 29, 44)
	Preparedness and willingness towards cultural change (professional culture)	1 (6.25)	(29)
	Expectations of users	3 (18.75)	(29, 38, 41)
	In general	6 (37.5)	(3, 17, 29, 38, 41, 44)
**Management**	Managers commitment	5 (31.25)	(29, 38, 40, 41, 44)
	Formulation and expression of a clear vision for the enterprise showing the HIS as part of it	1 (6.25)	(44)
	Setting clear goals and instructions	3 (18.75)	(3, 17, 44)
	Flexible planning	3 (18.75)	(29, 41, 44)
	Prospective and proactive control	1 (6.25)	(29)
	Coping with the impact of change	3 (18.75)	(3, 28, 44)
	Internal communication and clear feedback	3 (18.75)	(28, 41, 44)
	Having a strategy	4 (25)	(28, 40, 41, 44)
	Handling the diversity within stakeholder goals	2 (12.5)	(28, 38)
	Using formal project management methodology	2 (12.5)	(38, 46)
	Dedicate, availability and prioritize of competitive hospital resources (human, financial and physical resources and time)	6 (37.5)	(3, 17, 38, 41, 43, 44)
	Identify and mitigate risk (risk management)	1 (6.25)	(17)
	Consider IT implementation as a change process	1 (6.25)	(28)
	Understanding socio-technical nature of HISs	3 (18.75)	(11, 15, 17)
	Regular evaluations and using their results at different stages of HIS life cycle	2 (12.5)	(40, 44)
	In general	11 (68.75)	(3, 11, 15, 17, 28, 38, 40, 41, 43, 44, 46)
**Technical (system quality, information quality and service quality)**	Integration with Legacy system	3 (18.75)	(28, 40, 44)
	Interoperability and Interconnectivity	2 (12.5)	(17, 42)
	Usability	7 (43.75)	(28, 38, 40-42, 44, 46)
	Balance between flexibility and stability of IT	2 (12.5)	(28, 40)
	Reliable technical infrastructure or network,	2 (12.5)	(3, 38)
	Complexity of the system	3 (18.75)	(17, 28, 38)
	Information quality (relevancy, usefulness, completeness, etc.)	8 (50)	(3, 11, 15, 23, 28, 41, 42, 46)
	Response time (system speed)	5 (31.25)	(28, 40-42, 44)
	System security	3 (18.75)	(40-42)
	Service quality (the support provided by the information department, the support provided by the maintenance company)	5 (31.25)	(28, 40-43)
	Quality of user documentation	2 (12.5)	(40, 42)
	Flexibility and adoptability, enabling future functional and technical changes	5 (31.25)	(28, 38, 41, 42, 44)
	Using proper standards, coding and nomenclature	1 (6.25)	(40)
	In general	13 (81.25)	(3, 11, 15, 17, 23, 28, 38, 40-44, 46)
**Strategy**	National, regional, organizational	2 (12.5)	(28, 41)
	Accepted also at lower levels	1 (6.25)	(28)
	Alignment between system strategies and hospital strategies	3 (18.75)	(3, 38, 46)
	In general	7 (43.75)	(3, 28, 38, 40, 41, 44, 46)
**Economy**	Return on investment (material or immaterial)	4 (25)	(15, 28, 40, 41)
	Justification of increase of costs	4 (25)	(15, 28, 41, 45)
	Sufficient funding	4 (25)	(28, 40, 41, 44)
	In general	11 (68.75)	(3, 11, 15, 17, 28, 40, 41, 43-46)
**Education**	Sufficient training to make the best out of the daily operation	3 (18.75)	(17, 28, 40)
	Sufficient training to provide an understanding of its limitations and future potentials	1 (6.25)	(17)
	In general	7 (43.75)	(17, 28, 40-44)
**Legal**	Compliance with legal requirements	1 (6.25)	(28)
	Know what the legal constraints/opportunities are	1 (6.25)	(28)
	In general	2 (12.5)	(28, 38)
**Ethical**	Compliance with existing ethical rules in affairs management	1 (6.25)	(28)
	Privacy and confidentiality	1 (6.25)	(38)
	In general	3 (18.75)	(28, 38, 41)
**Political**	Political games/conflicts	2 (12.5)	(28, 38)
	Willingness towards investment on IT systems	1 (6.25)	(28)
	Reliable external partners	2 (12.5)	(28, 38)
	In general	5 (31.25)	(3, 28, 38, 41, 44)

**Table 4.** The HIS Evaluation Methods and Their Frequency in Selected Studies

Evaluation Methods	Frequency (%)	Reference No.
**Analysis of work procedure**	1 (6.25)	(47)
**Stakeholder analysis**	1 (6.25)	(47)
**Organizational readiness**	1 (6.25)	(44)
**Framework for assessment of strategies**	1 (6.25)	(47)
**Thinking-aloud**	2 (12.5)	(15, 45)
**Cognitive assessment**	2 (12.5)	(15, 47)
**Cognitive walkthrough**	2 (12.5)	(15, 47)
**Heuristic evaluation**	2 (12.5)	(15, 47)
**Balanced scorecard**	1 (6.25)	(47)
**Risk assessment**	3 (18.75)	(3, 40, 47)
**Focus group interview**	2 (12.5)	(40, 47)
**Delphi**	1 (6.25)	(47)
**Social network analysis**	2 (12.5)	(15, 47)
**Randomized controlled studies**	3 (18.75)	(15, 45, 47)
**Use proxy outcomes (decreased medication errors, improved adherence to practice guidelines, and improved quality of documentation)**	2 (12.5)	(45, 47)
**Interviews**	4 (25)	(23, 39, 45, 47)
**Questionnaires**	5 (31.25)	(11, 23, 39, 45, 47)
**Functionality assessment**	1 (6.25)	(47)
**Design-reality gap model**	1 (6.25)	(3)
**Logical framework approach (LFA)**	1 (6.25)	(47)
**Work sampling**	2 (12.5)	(23, 39)
**SWOT**	1 (6.25)	(47)
**Studying the existing documents and Chart review**	2 (12.5)	(23, 39)
**Technical verification**	2 (12.5)	(11, 47)
**Video recording**	3 (18.75)	(11, 45, 47)
**Time studies**	2 (12.5)	(23, 45)
**Kept the log**	2 (12.5)	(11, 23)
**Effect assessment**	1 (6.25)	(47)
**Field study**	1 (6.25)	(47)
**Cost minimization analysis**	1 (6.25)	(11)
**Cost effectiveness analysis**	3 (18.75)	(11, 40, 45)
**Cost utility analysis**	3 (18.75)	(11, 40, 45)
**Cost benefit analysis**	3 (18.75)	(11, 40, 45)

## 5. Discussion

The results of the present research indicate that the emphasis of HISs evaluation moves from technical subjects to human and organizational subjects and from objective to subjective issues. Therefore, this issue entails more familiarity with more qualitative evaluation methods.

It has been proved that the application of HISs can create basic changes in culture, policy and authority, which link professional groups to one another in an organization. However, these basic subjects have not been identified in many of success models. These issues result in the inability of these models to interpret some cases of failure (39). Many studies published about the success of information systems have been carried out by focusing on the model of Delone and McLean (48, 49). Since in this model some important factors have not been considered, the mentioned studies cannot present a comprehensive model in this field. For instance, they deal with supporting the top managers and the involvement of users, and point out that these factors can influence the level of success, but these variables are not considered in this model. Moreover, other factors like culture and organizational characteristics are not considered in this model. The changes of procedures and culture are among the obstacles which are reported in widespread use of health care information systems (41). Other studies emphasized on organizational and management factors such as management commitment, a champion and his/her characteristics (28, 38, 41, 51, 52). 

The findings indicate that, among different factors of success, user satisfaction to measure HISs success is of special significance (3, 11, 15, 17, 23, 28, 38-46). This factor is probably the most widespread factor used in measurement of success (11, 41, 42). Even though some researchers may doubt about the reliability and appropriateness of using the user satisfaction to measure the success of information systems (52, 53), the researches which have been carried out, have proved that satisfaction is a useful criterion to measure the success of the system (11, 32, 54, 55). However, in the field of health care information systems, only four percent of researches have used the criterion of the influence of user satisfaction, whereas this rate reaches almost 20 percent in the researches of information systems (56). The findings show that, in published literatures, special attention has been paid to the economy factors and their evaluation (3, 11, 15, 17, 28, 38, 40, 41, 43-46). On the other hand, when the efficiency of investment in IT is studied, some problems manifest themselves as follows (11):

1- The expenditures are mostly indirect, so their calculation is difficult

2- The benefits and organizational impacts are often intangible, so their realization may take a long time

In spite of these problems, findings show that there are appropriate and verified methods for evaluating economy aspects which provide useful information in this area. The other point which seems noticeable about the success factors of the HIS refers to two of selected articles indicating that the evaluation of HISs is one of the success factors (40, 44). We believe that the quality and quantity of evaluations and using their results at different stages of the HIS life cycle can be an influential and significant factor in the success of such systems, and this factor can be studied through methods like interviews and studying the existing documents.

The findings reveal that, among the suggested methods for the evaluation of success or failure factors of HISs, using questionnaires (11, 23, 39, 45, 47) and interviews (23, 39, 45, 47) are more emphasized. Questionnaires are the best approved method for the evaluation of personal opinion, perception and attitude which is widely used in information systems and health researches. A comprehensive interview and focus group interview are effective on understanding how and why events have happened or will occur and on the perception of users about how affairs can be done more efficiently (39).

Among the evaluation methods extracted for this research, some methods are based on the retrospective nature (like functionality assessment). The other kinds such as balanced scorecard and delphi methods might present a guideline for planning and revision HISs, which provide formative evaluation (57). Some methods may not meet the specific information needs in the related field completely, but can be used as valuable supportive means in the field of evaluation. As the symptoms of a disease comprise a part of the pattern of that disease all these methods present a pattern to show the success or failure (8). The presented methods are adapted from different sciences including psychology, social science, computer and health informatics sciences. Applying adapted evaluation methods requires methodological skills, discipline, innovation and flexibility to adapt the chosen method with the intended case, its situation and specifically information needs (8, 24).

 The major limitations of this study are that some selected studies deal with only success and failure factors (28, 38, 42, 43, 46), or focused on evaluation methods (47), but, due to the significance of these studies, they are considered and reviewed in this research. In addition, among the extracted factors, there were some factors which could be placed in different groups and several discussion sessions took place to choose the most appropriate category for each factor. Another limitation is that only the articles written in English were selected; therefore, there might be some noteworthy articles in this field published in other languages.

In most of the reviewed articles, the main focus has been laid on the necessity of using multi-method approaches and combining methods to obtain more comprehensive and useful results. The integrating of different methods can be beneficial to find an inclusive answer to evaluation questions. The integrity of supplementary methods, data sources, theories and observers are studied under the term of triangulation (58). The combination of qualitative data gathering approaches (such as observations and interviews) and quantitative data gathering approaches (such as questionnaires and work sampling) provides a good opportunity through triangulation to improve the quality of results (59).

Finally, in this research, the extraction of key concepts of each study was carried out through applying meta synthesis, and such concepts were put together and classified, then the suggested framework was formed to evaluate the HIS success [ Table 5 ]. This framework includes 12 main factors, 67 sub factors, and 33 suggested methods for the evaluation of these sub factors. One can determine the appropriate method based on this information and with a general review of the listed methods and factors, or he/she can create better and more comprehensive methods based on his/her information needs. Of course, more attempts and investigations are still necessary to be done in this field. 

**Table 5.** The HIS Success Factors and Their Suggested Evaluation Methods

Success Factors	Sub Factors	Evaluation Methods
**Functional**	Preparation of the user requirements	Analysis of work procedure, Stakeholder analysis, Organizational readiness, Framework for assessment of strategies
	Alignment of the role and design of the HIS (Task-technology adaption)	Thinking-aloud, Cognitive assessment, Cognitive walkthrough, Heuristic evaluation, Analysis of work procedure
	Flexibility towards dynamic changes and changes in the organizational context	Organizational readiness, Balanced scorecard, Risk assessment
	Added functionality are provided by the HIS, enabling users to provide new or better services	Focus group interview, Delphi, Social network analysis, Stakeholder analysis
	Improve clinical performance and outcomes	Randomized controlled studies, Use proxy outcomes (decreased medication errors, improved adherence to practice guidelines, and improved quality of documentation)
**Organizational**	Collaboration and cooperation	Social network analysis, Stakeholder analysis
	Participation in decision-making	Focus group interview
	Work from the workflow	Social network analysis
	Support from higher level organizations	Stakeholder analysis, Interviews (non-standardized)
	Make implementation a transparent process within the organization	Focus group interview, Social network analysis, Stakeholder analysis
	Organizational stability	Social network analysis, Organizational readiness
	Rate of hospital independence and authority	Interviews
	Organizational capacity for changes	Organizational readiness
**Behavioral**	User involvement	Social network analysis, Stakeholder analysis, Focus group interview, Questionnaires
	User engagement and commitment	Focus group interview, Social network analysis, Stakeholder analysis
	Resistance to changes	Organizational readiness
	User knowledge and skills	Focus group interview, Questionnaires
	Stakeholder, user and patient satisfaction	Focus group interview, Questionnaires
	Motivational activities	Focus group interview, Personal interviews
	User acceptance (perceived system ease of use, perceived system usefulness)	Focus group interview, Questionnaires
**Cultural**	Understand health care as a specific culture	Analysis of work procedure
	Understand the local culture (such as attention to cultural differences between public and private hospitals as well as developing and developed countries)	Analysis of work procedure, Functionality assessment, Design—reality gap model
	Preparedness and willingness towards cultural change (professional culture)	Organizational readiness
	Expectations of users	Focus group interview
**Management**	Managers commitment	Logical framework approach (LFA)
	Formulation and expression of a clear vision for the enterprise showing the HIS as part of it	Balanced scorecard, Framework for assessment of strategies
	Setting clear goals and instructions	Balanced scorecard, Framework for assessment of strategies
	Flexible planning	Documents and chart review
	Prospective and proactive control	Logical framework approach (LFA)
	Coping with the impact of change	Organizational readiness, Work sampling
	Internal communication and clear feedback	Social network analysis
	Having a strategy	Framework for assessment of strategies
	Handling the diversity within stakeholder goals	Stakeholder analysis, Organizational readiness
	Using formal project management methodology	Analysis of work procedure, Balanced scorecard, Framework for assessment of strategies
	Dedicate, availability and prioritize of competitive hospital resources (human, financial and physical resources and time)	Risk assessment, SWOT assessment
	Identify and mitigate risk (risk management)	Risk assessment, Design—reality gap model
	Consider IT implementation as a change process	Analysis of work procedure, Framework for assessment of strategies
	Understanding socio-technical nature of HISs	Interviews, Questionnaires, Delphi
	Regular evaluations and using their results at different stages of HIS life cycle	Interviews, Studying the existing documents and Chart review
**Technical (system quality, information quality and service quality)**	Integration with Legacy system	Technical verification
	Interoperability and Interconnectivity	Technical verification
	Usability	Cognitive assessment, Cognitive walkthrough, Heuristic evaluation, Video recording, Thinking-aloud, Work sampling, Time studies, Kept the log
	Balance between flexibility and stability of IT	Organizational readiness against change
	Reliable technical infrastructure or network,	Technical verification
	Complexity of the system	Technical verification
	Information quality (relevancy, usefulness, completeness, etc.)	Chart review, Questionnaires
	Response time (system speed)	Questionnaires, Time and motion studies, Work sampling
	System security	Technical verification
	Service quality (the support provided by the information department, the support provided by the maintenance company)	Review of contracted task agreement and chart review, Questionnaires, Interviews
	Quality of user documentation	Thinking-aloud, Questionnaires, Interviews
	Flexibility and adoptability, enabling future functional and technical changes	Technical verification
	Using proper standards, coding and nomenclature	Technical verification
**Strategy**	National, regional, organizational	Framework for assessment of strategies
	Accepted also at lower levels	Focus group interview
	Alignment between system strategies and hospital strategies	Framework for assessment of strategies
**Economy**	Return on investment (material or immaterial)	Delphi, Effect assessment, Field study, Cost minimization analysis, Cost effectiveness analysis, Cost utility analysis, Cost benefit analysis
	Justification of increase of costs	Delphi, Cost effectiveness analysis, Cost utility analysis, Cost benefit analysis
	Sufficient funding	Delphi, Cost effectiveness analysis, Cost utility analysis, Cost benefit analysis
**Education**	Sufficient training to make the best out of the daily operation	Functionality assessment, Work sampling, Time studies
	Sufficient training to provide an understanding of its limitations and future potentials	Focus group interview
**Legal**	Compliance with legal requirements	Field study, Review of legal documents
	Know what the legal constraints/opportunities are	SWOT, Interviews
**Ethical**	Compliance with existing ethical rules in affairs management	Focus group interview
	Privacy and confidentiality	Focus group interview, Chart review
**Political**	Political games/conflicts	SWOT, Delphi
	Willingness towards investment on IT systems	Interviews, Questionnaires
	Reliable external partners	Chart review and review of contracts as well as history of partners activities
